# The Link between Circadian Clock Genes and Autophagy in Chronic Obstructive Pulmonary Disease

**DOI:** 10.1155/2021/2689600

**Published:** 2021-10-25

**Authors:** Yuedi Hu, Tiantian He, Jie Zhu, Xiaole Wang, Jiabing Tong, Zegeng Li, Jingcheng Dong

**Affiliations:** ^1^College of Integrated Chinese and Western Medicine, Anhui University of Chinese Medicine, No. 1, Qianjiang Road, Hefei City, Anhui Province, China; ^2^Institutes of Integrative Medicine, Fudan University, Shanghai, China; ^3^Institute of Traditional Chinese Medicine Prevention and Control on Respiratory Disease, Anhui Academy of Chinese Medicine, No. 117, Meishan Road, Hefei City, Anhui Province, China; ^4^Department of Respiratory Medicine, First Affiliated Hospital of Anhui University of Traditional Chinese Medicine, Meishan Road, Hefei City, Anhui Province, China

## Abstract

Chronic obstructive pulmonary disease (COPD), a progressive respiratory disease, is characterized by the alveolar epithelium injury and persistent airway inflammation. It is documented that oscillation and dysregulated expression of circadian clock genes, like Bmal1, Per1, and Per2, involved in COPD pathogenies, including chronic inflammation and imbalanced autophagy level, and targeting the associations of circadian rhythm and autophagy is promising strategies in the management and treatment of COPD. Herein, we reviewed the mechanisms of the circadian clock and the unbalance of the autophagic level in COPD, as well as the link between the two, so as to provide further theoretical bases for the study on the pathogenesis of COPD.

## 1. Introduction

The 2021 GOLD Guideline classifies COPD as one of the top three causes of death worldwide [1]. The injury of the alveolar epithelium and inflammatory stimulation induced by repeated cigarette smoke (CS) exposure are key pathological processes in the onset and development of COPD, while CS-induced circadian clock dysfunction in COPD may promote the progression of COPD through inflammatory responses and oxidative stress [2]. Hwang et al. found that CS-stimulated mice have significantly different lung functions during day and night [3], which may be related to rhythm changes of CS-mediated surfactant proteins and pulmonary inflammation [4]. Therefore, inflammatory changes induced by pulmonary clock dysfunction may explain COPD's aggravation in poor functioning lungs during nighttime and early morning. Meanwhile, autophagy, above or below the baseline, has been shown to be related to cellular inflammatory responses. Overactivated autophagy can induce allergic airway inflammation in a murine model of respiratory diseases by activating oxidative stress [5]. Conversely, the loss of autophagy-related proteins (e.g., Beclin1, LC3B, ATG5, ATG 4b, and ATG 7) exacerbate neutrophilic infiltration, activate inflammasome (e.g., CXCL1 and NLRP3), and facilitate significant alterations in proinflammatory cytokines [6, 7]. These observations prove that COPD involves circadian clock dysfunction and autophagy dysregulation, thus suggesting it is necessary to investigate the relationship between circadian clock and autophagy.

## 2. The Role of the Circadian Clock in the Regulation of COPD

**Figure 1:**
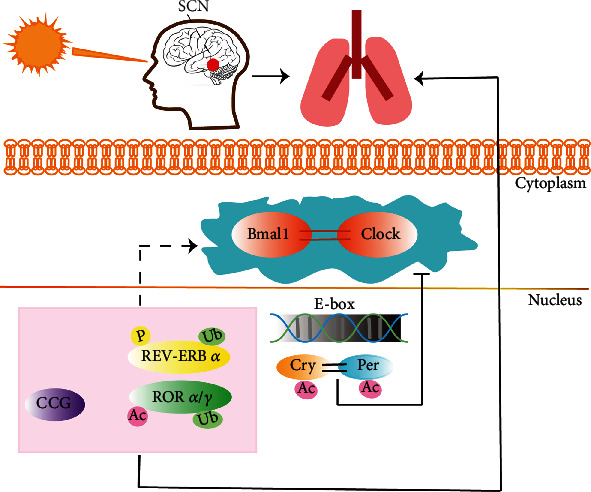
The regulation of circadian rhythm in the lung. The peripheral clock present in the lung is affected by SCN. In the form of a heterodimer, the clock/Bmal1 complex can activate the element sequence of E-box and regulate the expression of the Period family and Cryptochrome family. Per and Cry heterodimers are transmitted to the nucleus after being phosphorylated by casein kinases. They inhibit Per and Cry transcriptions by blocking the activity of the clock/Bmal1 complex. Nuclear receptors REV-ERB*α*/*β*, ROR*α*/*γ*, and CCG, are also involved in the expression of the circadian rhythm of their target genes by regulating transcription. CK: casein kinases; P: phosphorylate; Ub: ubiquitination; Ac: acetylation.

The brain's suprachiasmatic nucleus (SCN) receives electrical signals from the retina following light stimulation. Simultaneously, the effect of this photic information is synchronized to the oscillation of central clocks and transmits rhythmic information of SCN to peripheral organs and clocks [8]. Its operation is based on the feedback loop of the “transcription-translation” mechanism [9]. As the core factors of circadian rhythm, Bmal1 and clock have a crucial role in activating the feedback loop [10]. Heterodimers composed of Bmal1 and clock activate the element sequence of E-box and then regulate the transcription of genes [11]. By binding and activating in the E-box region, the complex of Bmal1 and clock (Bmal1/clock complex) regulates the expression of the Period (Per 1, 2, and 3) family and Cryptochrome (Cry 1 and 2) family. After the translation, the Per and Cry heterodimers are phosphorylated by casein kinases and are transmitted to the nucleus, where they inhibit Per and Cry transcription by blocking the activity of the Bmal1/clock complex [12]. In addition to Per/Cry, nuclear receptors REV-ERB*α*/*β* and ROR*α*/*γ* are also involved in the expression of the circadian rhythm of their target genes by regulating transcription [13]. Besides, the clock proteins are observably affected by posttranslational modifications. The modifications of posttranslation regulate protein renewal, intracellular localization, and DNA binding affinity and change the clock proteins' activity and stability through a series of circadian processes [14–16]. Meanwhile, the clock-controlled genes (CCG) are also affected by a molecular oscillator [12]. The abnormalities in clock gene function interfere with the timing and amplitude of CCG and the circadian processes they control and may lead to chronic diseases like asthma and Alzheimer's disease [16–19] (Figure 1).

Dysfunction of the circadian clock may promote COPD progression via inflammatory responses and oxidative stress [20]. Dysfunction of the circadian clock has been extensively studied in patients or experimental animals with lung diseases. In the lungs of mice with chronic CS exposure, the expression of Bmal1, REV-ERB*α*/*β* (Nr1d1), and Per1 decrease, and the express process of Per1 and Per2 may also change. Moreover, clinical studies have found a decrease in the number of Bmal1 in COPD patients' lungs. It is believed that such dysfunction partially results from clock's transcription regulated by acetylated and degraded Bmal1 and Per2 [3, 21]. Furthermore, the timing and amplitude of the circadian clock gene expression and the assumed impact on CCG expression are changed in rodents exposed to CS and proinflammatory mediators (e.g., LPS and TNF-*α*) [22]. According to the report of Yao et al. [21], the central and peripheral circadian clocks are involved in the bronchial epithelial cells (BECs) of mice and humans and the neutrophils of mice's bronchoalveolar lavage fluid. Consistently, these studies also demonstrated that the circadian clock could be modified by inflammation and regulate the timing of inflammatory responses. For example, the clock protein could bind to the nuclear transcription factor *κ*B (NF-*κ*B) and regulate its transcription activity [23]. These results suggest damaged oscillating timing, strength, and amplitude of circadian rhythm molecules in COPD that are accompanied by severer inflammation and oxidative stress.

## 3. Imbalanced Autophagy Level in COPD

Autophagy of physiological baseline can promote energy metabolism, stabilize the intracellular environment, eliminate pathogens, degrade damaged organelles, regulate inflammatory responses, and protect lung tissues [24–26]. For instance, the activation of autophagy-mediated by Wnt5A (one of the secreted Wnt glycoprotein ligands) signaling promotes the elimination of bacteria (*Pseudomonas aeruginosa* and *Streptococcus pneumoniae*) in macrophages [27]. According to a recent study, epithelial autophagy activated by AMPK has been observed in the lungs of developing mice, while the inhibition of AMPK-mediated autophagy led to reduced lung branching *in vitro* [28]. Importantly, both deficient and overactivated autophagy has been associated with the progression of COPD.

Long-lasting and repeated CS exposure results in impaired autophagy flux, possibly and partially due to the activation of the SIRT6-IGF-Akt-mTOR signaling pathway [2, 29]. CS exposure increases the expression of p62, the marker of impaired autophagy, and causes ubiquitinated proteins and the autophagy transcription factor TFEB to aggregate in insoluble protein fractions (aggresome bodies) [30]. These findings reveal the potential mechanism of impaired autophagy [31]. Furthermore, when the autophagy in BECs and lung homogenate of COPD patients are inhibited by 3MA (an autophagy chelation-specific inhibitor), cellular senescence and the secretion of inflammatory mediator interleukin-8 (IL-8) increases [29, 32].

Conversely, acute CS exposure triggers an increase in autophagic flux, which is accompanied by severer inflammation, and cellular senescence [30]. In BECs of mice, the expressions of LC3-II, Atg4B, Atg5-Atg12, and Atg7 significantly increased, autophagy vesicles were formed, and inflammatory cytokines (including TNF-*α*, IL-6, and IL-8) were upregulated [33, 34]. Takasaka et al. found that CS exposure promotes the autophagy activation of human bronchial epithelial cells (HBECs) and accelerates their aging by mediating the histone deacetylase SIRT6 [35]. CS induces autophagy by inhibiting mTOR (a negative regulator of autophagy) in epithelial cells of mice and humans. Mice with a knockout of *Mtor* in the bronchial or alveolar epithelial cells exhibited aggravated airway inflammation and enlarged airspace [36].

## 4. The Link between Clock Molecules and Autophagy in COPD

**Figure 2:**
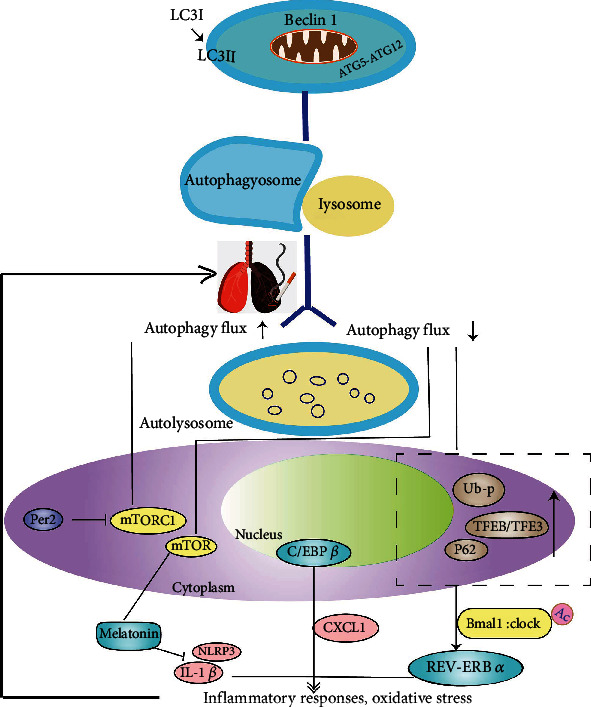
Circadian rhythm and autophagy regulation in COPD. The conversion of LC3-I to LC3-II is regarded as an indicator of autophagosome formation [47]. Meanwhile, autophagy proteins BECLIN 1 and ATG5-ATG12 are also involved in the formation of autophagosomes [48]. Autophagosome and lysosome form autolysosome under the digestion of lysosomal hydrolase [49]. CS exposure breaks the balance of the autophagy level and promotes COPD progression by enhancing inflammation and oxidative stress. The core clock protein Per2 can activate autophagy by specifically inhibiting the activity of mTORC1. mTOR in the macromolecular complex (mTOR complex 1 [(mTORC1)) negatively regulates autophagy and activates melatonin, while melatonin inhibits the NLRP3 inflammasome and IL-1*β* to alleviate airway inflammation. C/EBP*β* is involved in COPD development by participating in the expression of the inflammatory mediator CXCL1 in HBECs and the aggregation of neutrophils. CS exposure increases the expression of p62, inducing aggregation of ubiquitinated proteins and the TFEB in aggresome-bodies while participate in autophagy by regulating core clock genes. p62: the autophagy impairment marker; TFEB: autophagy transcription factor; REV-ERB*α* (Nr1d1): an important component in core circadian clock molecules; Ub-p, ubiquitinated protein.

The regulatory relationship between autophagy and circadian rhythm molecules was first discovered in the 1970s. Scientists found a diurnal rhythm of autophagic vacuolization and liver atrophy in meal-fed rats [37] and a circadian rhythm of autophagic core factors in drosophila, zebrafishes, and mammals [38–40]. With the development of molecular biology, the mechanism of action between autophagy and circadian rhythm molecules was gradually revealed. Subsequent studies have shown that the autophagy level in different tissues of different species can be controlled by multiple clock genes [39, 41, 42], especially Per2, Bmal1, and clock, which correlate with autophagy. For example, Per2, as the scaffolding protein in the liver, inhibits the activity of mTORC1 via recruiting the tuberous sclerosis complex 1 (TSC1) to mTORC1 complex [43]. *In vitro* experiments showed that the transient overexpression of Per2 led to the downregulation of the PI3K Class1/Akt pathway and an elevation in the autophagy flux [44, 45]. In addition, clock mutants reduced the degradation of Bmal1 through the autophagic pathway via proteasomes and under high-fat feeding, indicating that core clock components are involved in autophagy regulation [46] (Figure 2).

The interrelation between clock molecules and autophagy in lung tissues has been demonstrated in another study. As a histone deacetylase, SIRT1 has been proved to be able to activate autophagy. Lower activity of SIRT1 in the lung epithelial cells and macrophages of humans and mice exposed to CS further induces highly acetylated Bmal1, Per2, and clock proteins or clock gene-related histones and thus promotes inflammation and cell aging [50, 51]. All such results prove that both circadian clock dysfunction and imbalanced autophagy can contribute to COPD's development by promoting inflammatory responses, oxidative stress, or cellular senescence.

### 4.1. REV-ERB*α* and TFEB/TFE3 Participate in the Rhythmic Expression of Autophagy

REV-ERB*α* (Nr1d1) is an important component in core circadian clock molecules. Its expression follows the autophagy inhibition pattern basing on the circadian rhythm [52] and relates autophagy to the circadian clock. According to the research of Pariollaud et al., the expression of the REV-ERB*α* protein in the lung follows the diurnal variation. When REV-ERB*α* is knocked out, the rhythmic diurnal variation disappears, while inflammatory cytokines significantly increase in the lung of mice [53]. These data indicate that circadian clock molecules may mediate REV-ERB*α* to promote inflammatory responses and exacerbating COPD's progression. Furthermore, TFEB and TFE3, which are the key regulatory factors for autophagy, lysosomal biogenesis, and cellular homeostasis with circadian rhythm, regulate circadian clock genes involved in autophagy and the expression of REV-ERB*α* (Nr1d1) [54]. Enrichment of site-binding TFE3 and REV-ERB*α* is observed in promoter regions of genes (e.g., LAMP1, Mcoln1, Vps33a, Atg3, ATG5, CTSL, and Gabarapl1) involved in the circadian rhythm, autophagy, and lysosomal biogenesis. Meanwhile, the ChIP analysis from Pastore et al. showed several ChIP-seq peaks of TFEB or TFE3 in the promoter of REV-ERB*α* [54]. Inflammation-oxidative stress promoted by autophagy impairment mediated by perinuclear aggregation of TFEB is one of the significant pathological features of CS-induced COPD-emphysema [31]. This evidence suggests that TFEB or TFE3 (TFEB/TFE3) can bind to REV-ERB*α* to participate in autophagy's rhythmic expression and thus regulate pulmonary homeostasis [55].

### 4.2. C/EBP*β* Integrates the Rhythmic Expression of Autophagy Genes

As a nuclear transcription factor, C/EBP*β* participates in coordinating the rhythmic expression of autophagy genes. In physiological regulation, C/EBP*β* integrates the signals from the circadian rhythm to autophagy. For example, Ma et al. proved the rhythmic induction of C/EBP*β* in autophagy genes (e.g., Ulk1, Gabarapl1, LC3B, and Bnip3) in the liver and found that autophagy was related to the circadian clock and maintained the dynamic balance of nutrients throughout the light/dark cycle [55]. In pathological regulation, C/EBP*β*, which has a notable relation with diurnal rhythm, has been proved to be involved in COPD regulation by participating in the expression of the inflammatory mediator CXCL1 in HBECs and the aggregation of neutrophils [56].

### 4.3. Melatonin Can Inhibit Autophagy by Acting against Inflammation and Antioxidants

As an endocrine hormone, melatonin has been synthesized and secreted by the pineal gland in the brain. It participates in maintaining the normal operation of the circadian clock and regulating autophagy precursors to significantly enhance its protective effect on different systems, including the central nervous system, endocrine system, cardiovascular system, gastrointestinal system, and respiratory system [57–59]. It has been defined that melatonin strongly acts against inflammation and oxidation [60, 61]. Autophagy is believed to be an induced response to oxidative stress [7]. Accordingly, the antioxidant activity of melatonin can be explained as its inhibition against autophagy [62], which appears to be realized through mTOR activation and the JNK/Bcl-2/Beclin1 signaling pathway [63, 64]. Melatonin is known to inhibit ciclosporin-induced autophagy in rat pituitary GH3 cells through the MAPK/ERK pathway, which is completely or partially attributed to its antioxidant property [58]. Specifically, in rats with COPD, melatonin can inhibit the NLRP3 inflammasome and IL-1*β*, thus alleviating airway inflammation [62], suggesting that melatonin can delay the onset and development of COPD by acting against inflammation.

### 4.4. Circadian Clock Molecules Regulate mTORC1 in Two Ways

mTOR, a negative regulator of autophagy, is potentially involved in the inflammatory responses of pulmonary diseases. When the expression of mTOR is downregulated, the inflammation of COPD is activated, resulting in emphysema [65]. Existing studies have proved that the mTOR can be regulated by the circadian clock genes in two ways: (1) the core clock protein Per2 can inhibit the activity of mTORC1 from activating autophagy [39] and (2) circadian clock-related transcription factors positively regulate mTORC1. For instance, REV-ERB *α* can activate the mTORC1 pathway to inhibit autophagy and phosphorylation of the clock protein Bmal1 [66], and similarly, TFEB can activate mTORC1 by inducing cellular endocytosis and formation the endosomes carrying lysosomes [67].

## 5. Conclusion and Perspectives

Few studies have suggested that both autophagy and the circadian clock play an important role in COPD's pathogenesis. However, existing reports have provided possible insights and clues for the interaction between the two. Inflammatory responses and oxidative stress in COPD are the most common factors that lead to imbalanced autophagy and circadian rhythm dysfunction. They are also the main pathological cause of imbalanced autophagy and circadian rhythm dysfunction involved in COPD progression. In this review, we have discussed a possible link between the circadian clock and autophagy via intermediates, such as REV-ERB*α*, C/EBP*β*, mTORC1, and melatonin, which may all promote COPD progression by inducing inflammatory responses. The specific mechanism of the interrelation between the circadian clock and autophagy in COPD will be further investigated in future studies to provide theoretical bases for the study on COPD's pathogenesis.
